# Selinexor and Venetoclax Combination in Patients With Relapsed or Refractory Acute Myeloid Leukemia

**DOI:** 10.1002/ajh.70266

**Published:** 2026-03-08

**Authors:** Somedeb Ball, Farrukh T. Awan, Ben K. Tomlinson, Tess Stopczynski, Melissa A. Fischer, Zhiguo Zhao, Kateryna Fedorov, Ashwin Kishtagari, Sanjay R. Mohan, Gregory D. Ayers, Michael T. Byrne, Michael R. Savona

**Affiliations:** ^1^ Vanderbilt Ingram Cancer Center, Vanderbilt University Medical Center Nashville Tennessee USA; ^2^ Division of Hematology and Oncology, Department of Internal Medicine Vanderbilt University School of Medicine Nashville Tennessee USA; ^3^ Division of Hematology and Oncology University of Texas Southwestern Medical Center Dallas Texas USA; ^4^ Department of Hematology and Stem Cell Transplant University Hospitals Seidman Cancer Center Cleveland Ohio USA; ^5^ Department of Biostatistics Vanderbilt University School of Medicine Nashville Tennessee USA; ^6^ Tennessee Oncology Nashville Tennessee USA; ^7^ Program in Cancer Biology, Vanderbilt University School of Medicine Nashville Tennessee USA

## Abstract

Preclinical studies showed a synergistic antileukemia activity with combination of selective XPO1 inhibitor selinexor (SEL) and venetoclax (VEN), with potential to overcome VEN resistance by reducing the anti‐apoptotic protein MCL1. In an investigator‐sponsored, open‐label, phase Ib study (NCT03955783), adult patients with relapsed or refractory acute myeloid leukemia (R/R AML) were enrolled. After the dose‐escalation phase, SEL 80 mg po weekly plus VEN 400 mg/day following ramp‐up was deemed the recommended phase II dose. Responses were assessed with IWG2003 and ELN 2022 criteria. Nineteen patients with R/R AML were enrolled. Median age at enrollment was 67.2 (range, 21.1–83.8) years. Overall, patients received median of 3 (range, 1–5) prior lines of therapy. The most common grade 3–5 treatment emergent adverse events (TEAE) were anemia (39%), neutropenia (33%), febrile neutropenia (28%), and thrombocytopenia (28%). Overall, the response rate with SEL‐VEN was 21%. Two (11%) patients, one with prior allo‐HSCT and one with prior VEN and both treated with SEL 80 mg/week, experienced complete remissions, with duration of response of 7 and 9.1 months, respectively. After a median follow up of 3.0 (range, 0.6–15.4) months, median event‐free survival was 2.4 (95% CI: 1.9–12.1) months and median overall survival was 6.4 (95% CI: 2.5–12.1) months. In conclusion, SEL‐VEN was feasible and active in a heavily pretreated AML cohort, with no new toxicity signal, but survival outcomes remained poor. The second‐generation XPO1‐inhibitor eltanexor, combined with VEN may further improve outcomes in VEN resistant AML in an ongoing study (NCT06399640).

Despite advances in frontline regimens for acute myeloid leukemia (AML), the majority of patients with relapsed or refractory (R/R) AML continue to experience dismal outcomes [1]. The use of venetoclax (VEN), an inhibitor of anti‐apoptotic protein B cell lymphoma 2 (BCL2), has shown modest activity in early clinical studies in R/R AML. However, the duration of response and overall survival (OS) are short, highlighting the urgent need for novel therapeutic strategies for this patient population [2, 3]. VEN resistance is typically mediated by the emergence or expansion of specific somatic mutations (e.g.,*TP53 and RAS*) and upregulation of alternate anti‐apoptotic proteins, such as myeloid cell leukemia‐1 (MCL1) [4, 5]. Selective blockade of exportin‐1 (XPO1)/eukaryotic translation initiation factor (elF4E)‐mediated nuclear‐cytoplasmic transport results in nuclear retention and activation of tumor suppressor proteins (e.g., p53) and impaired translation of oncogenic proteins (e.g., MCL1) to promote selective cytotoxicity in cancer cells [6, 7]. Recent studies also highlight potential role of XPO1 in the disruption of leukemogenic condensates which regulate gene transcription [8, 9]. Selinexor (SEL/KPT‐330), a first‐in‐class XPO1‐inhibitor, has potent in vitro and in vivo antileukemia activity, including potential cytotoxicity for drug‐resistant leukemia initiating cells [10]. In a phase I clinical trial of SEL monotherapy in R/R AML, the objective response rate was 14%, with 31% of patients having a significant reduction in bone marrow blasts [11]. Our preclinical studies showed synergistic antileukemia activity of SEL‐VEN combination with the potential to overcome VEN resistance by MCL1 downregulation in R/R AML [5, 7].

In this investigator‐sponsored, multicenter, open‐label, phase Ib study (ClinicalTrials.gov identifier: NCT03955783), patients with high‐risk hematologic malignancies were enrolled at four US comprehensive cancer centers. The study included a dose escalation phase (standard “3 + 3” design), followed by a dose expansion phase (Simon's two stage optimal design). Adult patients with R/R AML (WHO 2016) with adequate performance status (ECOG‐PS 0‐1) and organ function were eligible. Patients with relapsed disease ≥ 3 months after allogeneic hematopoietic stem cell transplant (allo‐HSCT) were eligible if they did not have active graft versus host disease. Prior VEN exposure was allowed only in the dose expansion phase. Three dose levels (60, 80, and 100 mg) of oral SEL were evaluated. VEN was given orally on a continuous once daily dosing schedule, following an initial 3‐day ramp‐up in cycle 1. Concomitant use of moderate CYP3A inhibitors was allowed, with appropriate dose reduction of VEN per guidance from US prescribing information. Unless contraindicated, all patients were required to be on dual anti‐emetics (5‐HT3 blocker and olanzapine) through the end of cycle 2. Disease assessments were performed with serial bone marrow aspiration and biopsies per protocol. The primary objectives of the dose escalation phase were to understand the safety profile and to find a recommended phase 2 dose (RP2D) for SEL with VEN. Treatment emergent adverse events (TEAE) were assessed using the Common Terminology Criteria for Adverse Events (CTCAE) v5.0. In the dose expansion phase, the primary objective was to assess the efficacy of the combination using the overall response rate (ORR) per International Working Group (IWG 2003) [12] criteria. Responses were recalculated per European Leukemia Net (ELN 2022) criteria. The ORR included complete remission (CR), CR with incomplete count recovery, CR with partial hematologic recovery, partial remission (PR), and morphologic leukemia free state (MLFS). We assumed a historical ORR of 20% based on VEN monotherapy experience [2], and a target ORR of 40%, yielding a type I error rate of 5% and a power of 80%. Secondary objectives included the estimation of event‐free survival (EFS) and OS with the Kaplan–Meier method. Descriptive statistics were used to summarize outcome measures among subgroups.

Between September 2019 and December 2022, 19 patients with R/R AML were enrolled. Median age at enrollment was 67.2 (range, 21.1–83.8) years (Table 1). Overall, 53% of patients were male, 95% were White, and all were non‐Hispanic. Median prior lines of therapy were 3 (range, 1–5), including 8 (42%) with prior allo‐HSCT and 8 (42%) patients with prior VEN exposure. One‐third of patients had adverse‐risk cytogenetics (ELN 2022). Common mutations on targeted DNA‐sequencing before enrollment (*n* = 13) were *NPM1* (*n* = 3; 23%), *IDH2* (*n* = 3; 23%), *U2AF1* (*n* = 3; 23%), *NRAS* (*n* = 2; 15%), and *TP53* (*n* = 2; 15%). Patients received a median of 3 (range, 1–8) cycles of SEL‐VEN, with a median treatment duration of 1.9 (range, 0.3–8.1) months. Seven (37%) patients were treated in SEL 60 mg/week, and the remaining 12 patients (5 in dose escalation and 7 in dose expansion phases) in 80 mg/week dose cohorts. Based on overall safety and preliminary clinical activity, SEL dose of 80 mg/week was determined to be the RP2D. By data cut‐off, all patients had discontinued the study treatment, primarily due to disease progression (*n* = 9; 47%) (Figure S1). No dose limiting toxicity (DLT) was observed in this study population. Overall, 17 (94%) patients experienced a grade ≥ 3 TEAE (Table 2), with most common being anemia (39%), neutropenia (33%), febrile neutropenia (28%), and thrombocytopenia (28%). Common non‐hematologic TEAEs (mostly grade 1–2) included vomiting (50%), nausea (44%), anorexia (44%), diarrhea (39%), and fatigue (33%). Grade 3–4 non‐hematologic AEs included nausea, vomiting, anorexia, and hyponatremia (1 patient each) (Table 2; Table S1). Adverse events led to the discontinuation of study treatment in 3 (16%) patients. A serious adverse event (SAE) was noted in 13 (72%) patients (Table S2). The 30‐day and 60‐day mortality rates were 5% and 21% in our study population. The TEAEs led to death in 3 patients (fever, respiratory failure, and intracranial hemorrhage) on study, all deemed related to AML.

**TABLE 1:** Baseline characteristics of study population (*N* = 19).

Age (years), median (range)		67.2 (21.1, 83.8)
Sex, no (%)		
Male		10 (53%)
Female		9 (47%)
Race, no (%)		
White		18 (95%)
Black		1 (5%)
Ethnicity, no (%)		
Non‐Hispanic		19 (100%)
ECOG‐PS, no (%)		
0		5 (26%)
1		14 (74%)
Prior lines of therapy, median (range)		3 (1–5)
Prior venetoclax exposure, no (%)		8 (42%)
Prior Allo‐HSCT, no (%)		8 (42%)
Cytogenetic risk category (ELN 2022), no (%)^a^		
Favorable		1 (6%)
Intermediate		11 (61%)
Adverse		6 (33%)
Baseline mutations, no (%)^a^		
*NPM1*		3 (23%)
*IDH2*		3 (23%)
*U2AF1*		3 (23%)
*DNMT3A*		2 (15%)
*NRAS*		2 (15%)
*TP53*		2 (15%)
*ASXL1*		1 (8%)
*BCOR*		1 (8%)
*SRSF2*		1 (8%)
*RAD21*		1 (8%)
*FLT3‐ITD*		1 (8%)
*WT1*		1 (8%)

Abbreviations: Allo‐HSCT, allogeneic hematopoietic stem cell transplant; ECOG, Eastern Cooperative Oncology Group; ELN, European Leukemia Net; no, number; PS, performance status.

^a^ Percentage calculated on number of evaluable cases.

**TABLE 2:** Common treatment emergent adverse events.

Adverse events	Dose escalation (*n* = 12)				Dose expansion (*n* = 6)^a^		All patients (*n* = 18)^a^	
	SEL 60 mg/week (*n* = 7)		SEL 80 mg/week (*n* = 5)		SEL 80 mg/week			
	All	Grade 3–5	All	Grade 3–5	All	Grade 3–5	All	Grade 3–5^b^
Any AEs	7 (100%)	7 (100%)	5 (100%)	4 (80%)	6 (100%)	6 (100%)	18 (100%)	17 (94%)
Hematologic AEs								
Platelet count decreased	2 (29%)	2 (29%)	2 (40%)	2 (40%)	1 (17%)	1 (17%)	5 (28%)	5 (28%)
Anemia	3 (43%)	3 (43%)	2 (40%)	1 (20%)	3 (50%)	3 (50%)	8 (44%)	7 (39%)
Neutrophil count decreased	2 (29%)	2 (29%)	2 (40%)	2 (40%)	2 (33%)	2 (3%)	6 (33%)	6 (33%)
White blood cell decreased	1 (14%)	0	1 (20%)	21 (20%)	2 (33%)	2 (33%)	3 (17%)	1 (6%)
Febrile neutropenia	3 (43%)	3 (43%)	0	0	1 (17%)	1 (17%)	5 (28%)	5 (28%)
Non‐hematologic AEs								
Vomiting	4 (57%)	0	3 (60%)	1 (20%)	2 (33%)	0	9 (50%)	1 (6%)
Nausea	3 (43%)	0	4 (80%)	1 (20%)	1 (17%)	0	8 (44%)	1 (6%)
Anorexia	4 (57%)	1 (14%)	2 (40%)	0	2 (33%)	0	8 (44%)	1 (6%)
Diarrhea	4 (57%)	0	1 (20%)	0	2 (33%)	0	7 (39%)	0
Fatigue	0	0	2 (40%)	1 (20%)	4 (67%)	0	6 (33%)	1 (6%)
Dyspnea	2 (29%)	1 (14%)	2 (40%)	1 (20%)	2 (33%)	0	6 (33%)	2 (11%)
Weight loss	1 (14%)	0	1 (20%)	0	2 (33%)	0	4 (22%)	0
Limb edema	3 (43%)	0	2 (40%)	0	0	0	5 (28%)	0
Dizziness	2 (29%)	0	2 (40%)	0	1 (17%)	0	5 (28%)	0
Cough	1 (14%)	0	3 (60%)	0	0	0	4 (22%)	0
Epistaxis	2 (29%)	1 (14%)	0	0	2 (33%)	1 (17%)	4 (22%)	2 (11%)
Generalized muscle weakness	1 (14%)	0	1 (20%)	1 (20%)	2 (33%)	0	4 (22%)	1 (6%)
Hyponatremia	0	0	1 (20%)	0	3 (50%)	1 (17%)	4 (22%)	1 (6%)
Dysgeusia	1 (14%)	0	2 (40%)	0	1 (17%)	0	4 (22%)	0
Sepsis	0	0	1 (20%)	1 (20%)	1 (17%)	1 (17%)	2 (11%)	2 (11%)
Rash	1 (14%)	1 (14%)	1 (20%)	1 (20%)	0	0	2 (11%)	2 (11%)
Disease progression	1 (14%)	1 (14%)	1 (20%)	1 (20%)	0	0	2 (11%)	2 (11%)

Abbreviations: AE, adverse event; SEL, selinexor.

^a^ One patient did not have adverse event information.

^b^ G5 TEAEs in 3 patients (fever, respiratory failure, and intracranial hemorrhage), all deemed related to AML.

The ORR with SEL‐VEN combination was 21%, by both IWG 2003 and ELN 2022 criteria (Figure 1A). Six (32%) patients were non‐evaluable for response, including four with no post‐treatment bone marrow biopsy, and two patients who did not receive protocol‐specified required doses of study treatment. Hence, the ORR was 31% in the efficacy‐evaluable population (*n* = 13). Two (11%) patients (01–114 and 01–123), treated with SEL 80 mg/week, experienced CR. Subject 01–114 had relapsed AML post allo‐HSCT with *BCOR*, *IDH2*, and *SRSF2* mutations, and had a duration of response of 7 months with SEL‐VEN. The other patient (01–123) with relapsed AML and t (16;16), trisomy 8, and a *WT1* mutation (detected post‐VEN exposure) experienced a CR with a duration of response of 9.1 months. One (5%) patient (02–203) experienced MLFS. Subject 01–104 with post allo‐HSCT R/R AML (mutations in *NPM1*, *IDH2*, *PTPN11*) had a medullary response (BM blast < 5% and satisfied all hematologic criteria of CR), but extramedullary disease detected at baseline persisted post‐treatment. Overall, the median number of cycles to best response was 2 (range, 2–4), and the median duration of response was 1.91 months (IQR: 1.87, 2.38). After a median follow up of 3.02 (range, 0.62–15.4) months, median EFS was 2.4 (95% CI: 1.9–12.1) months and median OS was 6.4 (95% CI: 2.5–12.1) months (Figure 1B,C).

**FIGURE 1:**
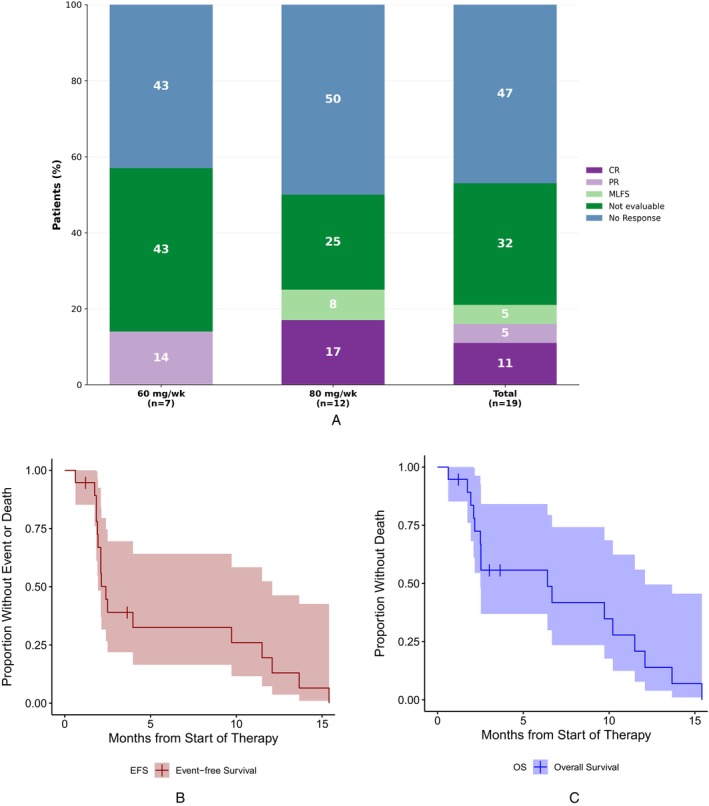
(A) Responses with selinexor and venetoclax in relapsed or refractory acute myeloid leukemia, (B) event‐free survival of study population, (C) overall survival of study population. [Color figure can be viewed at wileyonlinelibrary.com]

Patients with R/R AML continue to experience poor outcomes, with survival being particularly short in VEN‐resistant AML [13]. Despite strong preclinical evidence for MCL1 inhibition to overcome VEN resistance, clinical development of MCL1 inhibitors has been challenging due to on‐target cardiac toxicities [5, 14]. Hence, indirect MCL1 inhibition via modulation of other pathways (e.g., XPO1, CDK9) is being actively investigated in clinical studies [15]. We previously showed a dose‐dependent decrease in MCL1 and an increase in p53 levels following treatment of AML cell lines with XPO1‐inhibitors. Co‐treatment with XPO1‐inhibitor and VEN further lowered MCL1 levels, leading to a synergistic antileukemia activity. Interestingly, a high degree of synergy between XPO1 inhibitors and VEN was observed even in samples known to be VEN‐refractory in clinic [7]. Several other potential mechanisms involving modulation of DNA damage response and glycolytic metabolism have been proposed in support of XPO1 inhibitor‐VEN synergy [16, 17, 18]. Emerging evidence suggests that specific genomic subgroups, such as splicing factor mutated and menin‐dependent leukemias might be particularly sensitive to XPO1 inhibition [9, 19]. In our phase 1b trial with SEL‐VEN in R/R AML, grade ≥ 3 hematologic TEAEs were expectedly common (39%). Constitutional and gastrointestinal AEs were prevalent among non‐hematologic toxicities, although the majority were grade 1–2 and manageable with appropriate supportive measures. Importantly, two heavily pretreated patients experienced CR with SEL‐VEN, with clinically meaningful duration of response and survival. Two patients with post allo‐HSCT relapses had objective responses, suggesting potential SEL‐VEN activity in this particularly recalcitrant subgroup. Likewise, it was encouraging to see a durable CR in a subject with prior VEN exposure, which supports our preclinical data of SEL‐VEN activity in VEN‐refractory AML, an area of considerable unmet need in clinical practice. The findings of our study are limited by its small sample size and limited representation of different molecular subtypes of AML. The OS of study population was limited, as has been noted in preliminary clinical experience with azacitidine‐SEL‐VEN triplet [20], highlighting the molecular heterogeneity and diverse resistance mechanisms in R/R AML. Nevertheless, our study provides the early clinical evidence of safety, feasibility, and activity of XPO1‐inhibitor and VEN combination in R/R AML. Second‐generation XPO1‐inhibitor eltanexor with VEN could further improve outcomes in an ongoing study (NCT06399640) and provide a more tolerable XPO1‐inhibitor‐based regimen to overcome VEN‐resistant AML.

## Author Contributions

Conception and design: M.T.B., M.R.S.; Provision of study materials or patients: M.T.B., F.T.A., B.K.T., A.K., S.R.M., M.R.S.; Collection and assembly of data: S.B., M.T.B., K.F., M.A.F., T.S.; Data analysis and interpretation: S.B., T.S., G.D.A., Z.Z., M.R.S.; Manuscript writing: S.B., M.R.S.; Final approval of manuscript: All authors.

## Funding

This work was supported by Karyopharm Therapeutics.

## Ethics Statement

Ethical approval for this study was obtained from the Institutional Review Boards of all participating institutions.

## Consent

Written informed consent was obtained from all individual participants included in the study.

## Conflicts of Interest

Somedeb Ball, Ben K. Tomlinson, Tess Stopczynski, Melissa A Fischer, Zhiguo Zhao, Kateryna Fedorov, Gregory D. Ayers, and Michael T. Byrne: No conflicts of interest to disclose; Farrukh T. Awan: Loxo Oncology: Consultancy; ADC Therapeutics: Consultancy; Adaptive Biotechnologies: Consultancy; AbbVie/Pharmacyclics: Consultancy, Research Funding; BMS: Consultancy; Dava Oncology: Consultancy; Genmab: Consultancy; BeOne: Consultancy; Incyte: Consultancy; AstraZeneca: Consultancy; Miltenyi: Consultancy; Invivyd: Consultancy; Pierre Fabre: Consultancy; and received research funding from Actinium. Also serves on the DSMC for Ascentage, Astrazeneca, Caribou Biosciences. Tomlinson: BMS: Consultancy, Research Funding. Ashwin Kishtagari: Syndax: Current equity holder in publicly‐traded company; Sobi: Membership on an entity's Board of Directors or advisory committees, Speakers Bureau; Sevier Pharmaceuticals: Consultancy, Membership on an entity's Board of Directors or advisory committees; Morphosys: Membership on an entity's Board of Directors or advisory committees; Rigel: Membership on an entity's Board of Directors or advisory committees; Geron Coporation: Current equity holder in publicly‐traded company, Membership on an entity's Board of Directors or advisory committees. Sanjay R Mohan: Kartos: Research Funding; Karyopharm: Research Funding; Taiho: Research Funding; Ichnos: Research Funding; Incyte: Research Funding. Michael R. Savona: AbbVie; Bristol Myers Squibb; Calytrix, Geron; GSK, Karyopharm; Pharmaessentia, Ryvu Therapeutics: Consultancy; ALX Oncology Inc.; Astex Pharmaceuticals; Incyte Corporation; Prelude; and Takeda: Research Funding; Empath Biosciences; Karyopharm and Ryvu Therapeutics: Current holder of stock options; Astex Pharmaceuticals for travel grant. Other: Financial or Material Support.

## Supporting information


**Figure S1:** Patient enrollment and disposition (data cut‐off March 19, 2024).
**Table S1:** Summary of maximum grade adverse event by category and type for patients who experienced an adverse event (*n* = 18).
**Table S2:** Summary of serious adverse events in study population.

## Data Availability

The data that support the findings of this study are available on request from the corresponding author. The data are not publicly available due to privacy or ethical restrictions.

## References

[1] H. Kantarjian , G. Borthakur , N. Daver , et al., “Current Status and Research Directions in Acute Myeloid Leukemia,” Blood Cancer Journal 14, no. 1 (2024): 163. 39300079 10.1038/s41408-024-01143-2 PMC11413327

[2] M. Konopleva , D. A. Pollyea , J. Potluri , et al., “Efficacy and Biological Correlates of Response in a Phase II Study of Venetoclax Monotherapy in Patients With Acute Myelogenous Leukemia,” Cancer Discovery 6, no. 10 (2016): 1106–1117. 27520294 10.1158/2159-8290.CD-16-0313 PMC5436271

[3] C. D. DiNardo , A. Maiti , C. R. Rausch , et al., “10‐Day Decitabine With Venetoclax for Newly Diagnosed Intensive Chemotherapy Ineligible, and Relapsed or Refractory Acute Myeloid Leukaemia: A Single‐Centre, Phase 2 Trial,” Lancet Haematology 7, no. 10 (2020): e724–e736. 32896301 10.1016/S2352-3026(20)30210-6 PMC7549397

[4] C. D. DiNardo , I. S. Tiong , A. Quaglieri , et al., “Molecular Patterns of Response and Treatment Failure After Frontline Venetoclax Combinations in Older Patients With AML,” Blood 135, no. 11 (2020): 791–803. 31932844 10.1182/blood.2019003988 PMC7068032

[5] M. A. Fischer , Y. Song , M. P. Arrate , et al., “Selective Inhibition of MCL1 Overcomes Venetoclax Resistance in a Murine Model of Myelodysplastic Syndromes,” Haematologica 108, no. 2 (2023): 522–531. 35979721 10.3324/haematol.2022.280631 PMC9890032

[6] J. G. Turner , J. Dawson , C. L. Cubitt , R. Baz , and D. M. Sullivan , “Inhibition of CRM1‐Dependent Nuclear Export Sensitizes Malignant Cells to Cytotoxic and Targeted Agents,” Seminars in Cancer Biology 27 (2014): 62–73. 24631834 10.1016/j.semcancer.2014.03.001 PMC4108511

[7] M. A. Fischer , S. Y. Friedlander , M. P. Arrate , et al., “Venetoclax Response Is Enhanced by Selective Inhibitor of Nuclear Export Compounds in Hematologic Malignancies,” Blood Advances 4, no. 3 (2020): 586–598. 32045477 10.1182/bloodadvances.2019000359 PMC7013257

[8] F. Charles Cano , A. Kloos , R. Y. Hebalkar , et al., “XPO1‐Dependency of DEK:: NUP214 Leukemia,” Leukemia 39, no. 5 (2025): 1102–1113. 40148556 10.1038/s41375-025-02570-1 PMC12055596

[9] X. Q. D. Wang , D. Fan , Q. Han , et al., “Mutant NPM1 Hijacks Transcriptional Hubs to Maintain Pathogenic Gene Programs in Acute Myeloid Leukemia,” Cancer Discovery 13, no. 3 (2023): 724–745. 36455589 10.1158/2159-8290.CD-22-0424 PMC9975662

[10] J. Etchin , J. Montero , A. Berezovskaya , et al., “Activity of a Selective Inhibitor of Nuclear Export, Selinexor (KPT‐330), Against AML‐Initiating Cells Engrafted Into Immunosuppressed NSG Mice,” Leukemia 30, no. 1 (2016): 190–199. 26202935 10.1038/leu.2015.194 PMC4994896

[11] R. Garzon , M. Savona , R. Baz , et al., “A Phase 1 Clinical Trial of Single‐Agent Selinexor in Acute Myeloid Leukemia,” Blood 129, no. 24 (2017): 3165–3174. 28336527 10.1182/blood-2016-11-750158 PMC5524530

[12] B. D. Cheson , J. M. Bennett , K. J. Kopecky , et al., “Revised Recommendations of the International Working Group for Diagnosis, Standardization of Response Criteria, Treatment Outcomes, and Reporting Standards for Therapeutic Trials in Acute Myeloid Leukemia,” Journal of Clinical Oncology 21, no. 24 (2003): 4642–4649. 14673054 10.1200/JCO.2003.04.036

[13] A. Maiti , C. R. Rausch , J. E. Cortes , et al., “Outcomes of Relapsed or Refractory Acute Myeloid Leukemia After Frontline Hypomethylating Agent and Venetoclax Regimens,” Haematologica 106, no. 3 (2021): 894–898. 32499238 10.3324/haematol.2020.252569 PMC7927994

[14] ASH Clinical News , “FDA Places Trials of MCL‐1 Inhibitor on Clinical Hold,” 2019.

[15] A. H. Wei , A. W. Roberts , A. Spencer , et al., “Targeting MCL‐1 in Hematologic Malignancies: Rationale and Progress,” Blood Reviews 44 (2020): 100672. 32204955 10.1016/j.blre.2020.100672 PMC7442684

[16] J. Jiang , Y. Wang , D. Liu , et al., “Selinexor Synergistically Promotes the Antileukemia Activity of Venetoclax in Acute Myeloid Leukemia by Inhibiting Glycolytic Function and Downregulating the Expression of DNA Replication Genes,” ImmunoTargets and Therapy 12 (2023): 135–147. 38026089 10.2147/ITT.S429402 PMC10680489

[17] H. Yu , S. Wu , S. Liu , et al., “Venetoclax Enhances DNA Damage Induced by XPO1 Inhibitors: A Novel Mechanism Underlying the Synergistic Antileukaemic Effect in Acute Myeloid Leukaemia,” Journal of Cellular and Molecular Medicine 26, no. 9 (2022): 2646–2657. 35355406 10.1111/jcmm.17274 PMC9077288

[18] S. Yuan , W. Zuo , T. Liu , and H. Fu , “The Therapeutic Synergy of Selinexor and Venetoclax in Mantle Cell Lymphoma Through Induction of DNA Damage and Perturbation of the DNA Damage Response,” Technology in Cancer Research & Treatment 22 (2023): 15330338231208608. 37880950 10.1177/15330338231208608 PMC10605683

[19] S. Chaudhry , F. Beckedorff , S. S. Jasdanwala , et al., “Altered RNA Export by SF3B1 Mutants Confers Sensitivity to Nuclear Export Inhibition,” Leukemia 38, no. 9 (2024): 1894–1905. 38997434 10.1038/s41375-024-02328-1 PMC11347370

[20] L. N. Liu , Y. S. Cui , Y. Z. Liu , et al., “The Clinical Safety and Efficacy of Selinexor Combined With Venetoclax and Azactitidine Induction Therapy in Relapsed and Refractory Acute Myeloid Leukemia,” Zhonghua Xue Ye Xue Za Zhi 45, no. 8 (2024): 772–775. 39307725 10.3760/cma.j.cn121090-20231031-00241 PMC11535561

