# Supplementary material for: Selinexor and Venetoclax Combination in Patients With Relapsed or Refractory Acute Myeloid Leukemia
Source: Am J Hematol. 2026 Mar 8;101(5):1019–24. doi: 10.1002/ajh.70266 (PMC13055113; doi:10.1002/ajh.70266)
Supplement: Supplementary file 1 — Figure S1: Patient enrollment and disposition (data cut‐off March 19, 2024). Table S1: Summary of maximum grade adverse event by category and type for patients who experienced an adverse event (n = 18). Table S2: Summary of serious adverse events in study population. [file AJH-101-1019-s001.docx]

**
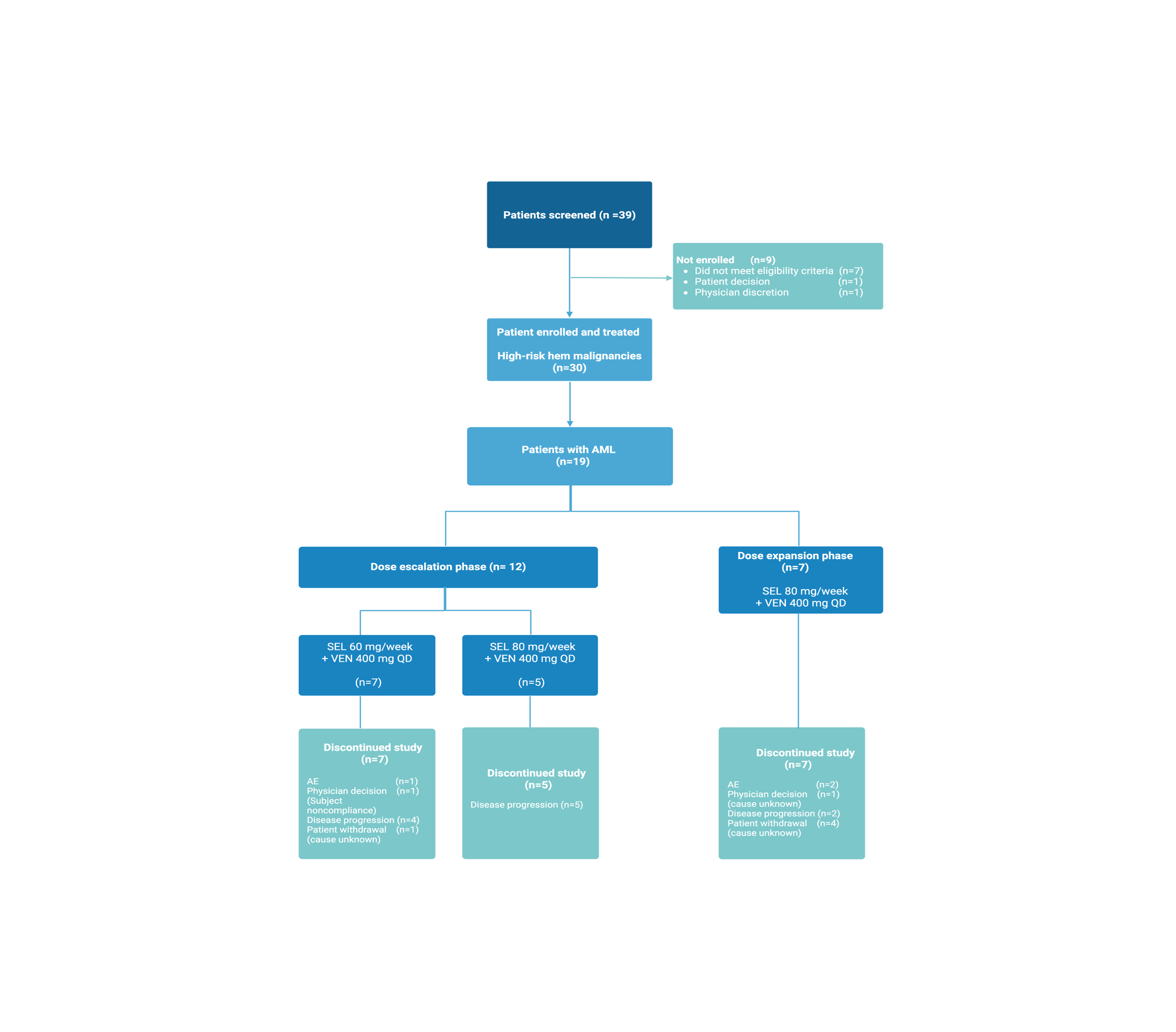
Supplementary Figure 1.** Patient enrollment and disposition (data cut-off March 19, 2024)

**Supplementary Table 1.** Summary of maximum grade adverse event by category and type for patients who experienced an adverse event (n=18).

| **Event category—*n* (%)** | **Total** | **Grade 1, 2** | **Grade 3, 4** | **Grade 5** |
| --- | --- | --- | --- | --- |
| **Blood and lymphatic system disorders, n=11** |  |  |  |  |
| Anemia | 8 (44.4) | 1 (5.6) | 7 (38.9) | 0 (0.0) |
| Febrile neutropenia | 5 (27.8) | 0 (0.0) | 5 (27.8) | 0 (0.0) |
| Other blood and lymphatic system disorder | 4 (22.2) | 1 (5.6) | 3 (16.7) | 0 (0.0) |
| **Gastrointestinal disorders, n=12** |  |  |  |  |
| Abdominal pain | 1 (5.6) | 1 (5.6) | 0 (0.0) | 0 (0.0) |
| Constipation | 2 (11.1) | 2 (11.1) | 0 (0.0) | 0 (0.0) |
| Diarrhea | 7 (38.9) | 7 (38.9) | 0 (0.0) | 0 (0.0) |
| Fecal incontinence | 1 (5.6) | 2 (11.1) | 0 (0.0) | 0 (0.0) |
| Other | 3 (16.7) | 3 (16.7) | 0 (0.0) | 0 (0.0) |
| Gingival pain | 1 (5.6) | 1 (5.6) | 0 (0.0) | 0 (0.0) |
| Hemorrhoids | 1 (5.6) | 1 (5.6) | 0 (0.0) | 0 (0.0) |
| Nausea | 8 (44.4) | 7 (38.9) | 1 (5.6) | 0 (0.0) |
| Vomiting | 9 (50.0) | 8 (44.4) | 1 (5.6) | 0 (0.0) |
| **Injury, poisoning and procedural complications, n=2** |  |  |  |  |
| Hip fracture | 1 (5.6) | 0 (0.0) | 1 (5.6) | 0 (0.0) |
| Other | 1 (5.6) | 1 (5.6) | 0 (0.0) | 0 (0.0) |
| **Investigations, n=9** |  |  |  |  |
| Aspartate aminotransferase increased | 1 (5.6) | 1 (5.6) | 0 (0.0) | 0 (0.0) |
| Blood bilirubin increased | 1 (5.6) | 1 (5.6) | 0 (0.0) | 0 (0.0) |
| Creatinine increased | 1 (5.6) | 1 (5.6) | 0 (0.0) | 0 (0.0) |
| Other | 5 (27.8) | 5 (27.8) | 0 (0.0) | 0 (0.0) |
| Lymphocyte count decreased | 3 (16.7) | 2 (11.1) | 1 (5.6) | 0 (0.0) |
| Neutrophil count decreased | 6 (33.3) | 0 (0.0) | 6 (33.3) | 0 (0.0) |
| Platelet count decreased | 5 (27.8) | 0 (0.0) | 5 (27.8) | 0 (0.0) |
| Weight loss | 4 (22.2) | 4 (22.2) | 0 (0.0) | 0 (0.0) |
| White blood cell decreased | 3 (16.7) | 0 (0.0) | 3 (16.7) | 0 (0.0) |
| **Nervous system disorders, n=10** |  |  |  |  |
| Cognitive disturbance | 1 (5.6) | 1 (5.6) | 0 (0.0) | 0 (0.0) |
| Dizziness | 5 (27.8) | 5 (27.8) | 0 (0.0) | 0 (0.0) |
| Encephalopathy | 1 (5.6) | 0 (0.0) | 1 (5.6) | 0 (0.0) |
| Headache | 1 (5.6) | 1 (5.6) | 0 (0.0) | 0 (0.0) |
| Intracranial hemorrhage | 1 (5.6) | 0 (0.0) | 0 (0.0) | 1 (5.6) |
| Memory impairment | 1 (5.6) | 1 (5.6) | 0 (0.0) | 0 (0.0) |
| Other | 1 (5.6) | 0 (0.0) | 1 (5.6) | 0 (0.0) |
| Peripheral sensory neuropathy | 2 (11.1) | 2 (11.1) | 0 (0.0) | 0 (0.0) |
| Somnolence | 2 (11.1) | 1 (5.6) | 1 (5.6) | 0 (0.0) |
| Tremor | 1 (5.6) | 1 (5.6) | 0 (0.0) | 0 (0.0) |
| **Skin and subcutaneous tissue disorders, n=5** |  |  |  |  |
| Dry skin | 2 (11.1) | 2 (11.1) | 0 (0.0) | 0 (0.0) |
| Pruritus | 1 (5.6) | 1 (5.6) | 0 (0.0) | 0 (0.0) |
| Purpura | 1 (5.6) | 1 (5.6) | 0 (0.0) | 0 (0.0) |
| Other | 3 (16.7) | 3 (16.7) | 0 (0.0) | 0 (0.0) |
| **Metabolism and nutrition disorders, n=12** |  |  |  |  |
| Alkalosis | 1 (5.6) | 1 (5.6) | 0 (0.0) | 0 (0.0) |
| Anorexia | 8 (44.4) | 7 (38.9) | 1 (5.6) | 0 (0.0) |
| Dehydration | 1 (5.6) | 1 (5.6) | 0 (0.0) | 0 (0.0) |
| Hyperglycemia | 1 (5.6) | 1 (5.6) | 0 (0.0) | 0 (0.0) |
| Hypoalbuminemia | 1 (5.6) | 1 (5.6) | 0 (0.0) | 0 (0.0) |
| Hypocalcemia | 2 (11.1) | 2 (11.1) | 0 (0.0) | 0 (0.0) |
| Hypokalemia | 1 (5.6) | 0 (0.0) | 1 (5.6) | 0 (0.0) |
| Hypomagnesemia | 2 (11.1) | 2 (11.1) | 0 (0.0) | 0 (0.0) |
| Hyponatremia | 4 (22.2) | 3 (16.7) | 1 (5.6) | 0 (0.0) |
| Hypophosphatemia | 2 (11.1) | 2 (11.1) | 0 (0.0) | 0 (0.0) |
| Other | 4 (22.2) | 3 (16.7) | 1 (5.6) | 0 (0.0) |
| **Musculoskeletal and connective tissue disorders, n=8** |  |  |  |  |
| Back pain | 1 (5.6) | 1 (5.6) | 0 (0.0) | 0 (0.0) |
| Bone pain | 2 (11.1) | 1 (5.6) | 1 (5.6) | 0 (0.0) |
| Generalized muscle weakness | 4 (22.2) | 3 (16.7) | 1 (5.6) | 0 (0.0) |
| Myalgia | 1 (5.6) | 1 (5.6) | 0 (0.0) | 0 (0.0) |
| Myositis | 1 (5.6) | 1 (5.6) | 0 (0.0) | 0 (0.0) |
| Neck pain | 1 (5.6) | 1 (5.6) | 0 (0.0) | 0 (0.0) |
| Pain in extremity | 1 (5.6) | 1 (5.6) | 0 (0.0) | 0 (0.0) |
| **Respiratory, thoracic, mediastinal disorders, n=12** |  |  |  |  |
| Aspiration | 1 (5.6) | 1 (5.6) | 0 (0.0) | 0 (0.0) |
| Atelectasis | 1 (5.6) | 1 (5.6) | 0 (0.0) | 0 (0.0) |
| Cough | 4 (22.2) | 4 (22.2) | 0 (0.0) | 0 (0.0) |
| Dyspnea | 6 (33.3) | 4 (22.2) | 2 (11.1) | 0 (0.0) |
| Epistaxis | 4 (22.2) | 2 (11.1) | 2 (11.1) | 0 (0.0) |
| Hypoxia | 4 (22.2) | 1 (5.6) | 3 (16.7) | 0 (0.0) |
| Pleural effusion | 1 (5.6) | 1 (5.6) | 0 (0.0) | 0 (0.0) |
| Pleuritic pain | 1 (5.6) | 1 (5.6) | 0 (0.0) | 0 (0.0) |
| Productive cough | 1 (5.6) | 1 (5.6) | 0 (0.0) | 0 (0.0) |
| Respiratory failure | 1 (5.6) | 0 (0.0) | 0 (0.0) | 1 (5.6) |
| Other | 6 (33.3) | 6 (33.3) | 0 (0.0) | 0 (0.0) |
| Wheezing | 1 (5.6) | 1 (5.6) | 0 (0.0) | 0 (0.0) |
| **General disorders and administration site conditions, n=14** |  |  |  |  |
| Chills | 2 (11.1) | 2 (11.1) | 0 (0.0) | 0 (0.0) |
| Edema limbs | 5 (27.8) | 5 (27.8) | 0 (0.0) | 0 (0.0) |
| Fatigue | 6 (33.3) | 5 (27.8) | 1 (5.6) | 0 (0.0) |
| Fever | 2 (11.1) | 1 (5.6) | 0 (0.0) | 1 (5.6) |
| Other | 9 (50.0) | 4 (22.2) | 2 (11.1) | 3 (16.7) |
| Pain | 1 (5.6) | 1 (5.6) | 0 (0.0) | 0 (0.0) |
| **Psychiatric disorders, n=3** |  |  |  |  |
| Insomnia | 2 (11.1) | 2 (11.1) | 0 (0.0) | 0 (0.0) |
| Other | 2 (11.1) | 2 (11.1) | 0 (0.0) | 0 (0.0) |
| **Eye disorders, n=4** |  |  |  |  |
| Blurred vision | 2 (11.1) | 2 (11.1) | 0 (0.0) | 0 (0.0) |
| Cataract | 1 (5.6) | 1 (5.6) | 0 (0.0) | 0 (0.0) |
| Other | 2 (11.1) | 2 (11.1) | 0 (0.0) | 0 (0.0) |
| **Infections and infestations, n=7** |  |  |  |  |
| Other | 4 (22.2) | 4 (22.2) | 0 (0.0) | 0 (0.0) |
| Lung infection | 1 (5.6) | 0 (0.0) | 1 (5.6) | 0 (0.0) |
| Sepsis | 2 (11.1) | 0 (0.0) | 2 (11.1) | 0 (0.0) |
| **Renal and urinary disorders, n=4** |  |  |  |  |
| Acute kidney injury | 1 (5.6) | 0 (0.0) | 1 (5.6) | 0 (0.0) |
| Hematuria | 1 (5.6) | 1 (5.6) | 0 (0.0) | 0 (0.0) |
| Other | 1 (5.6) | 1 (5.6) | 0 (0.0) | 0 (0.0) |
| Urinary retention | 1 (5.6) | 1 (5.6) | 0 (0.0) | 0 (0.0) |
| **Cardiac disorders, n=5** |  |  |  |  |
| Atrial fibrillation | 1 (5.6) | 0 (0.0) | 1 (5.6) | 0 (0.0) |
| Atrial flutter | 1 (5.6) | 1 (5.6) | 0 (0.0) | 0 (0.0) |
| Other | 1 (5.6) | 1 (5.6) | 0 (0.0) | 0 (0.0) |
| Sinus bradycardia | 1 (5.6) | 1 (5.6) | 0 (0.0) | 0 (0.0) |
| Sinus tachycardia | 3 (16.7) | 3 (16.7) | 0 (0.0) | 0 (0.0) |
| **Ear and labyrinth disorders, n=2** |  |  |  |  |
| Other | 1 (5.6) | 1 (5.6) | 0 (0.0) | 0 (0.0) |
| Vertigo | 1 (5.6) | 0 (0.0) | 1 (5.6) | 0 (0.0) |
| **Immune system disorders, n=2** |  |  |  |  |
| Other | 2 (11.1) | 1 (5.6) | 1 (5.6) | 0 (0.0) |
| **Vascular disorders, n=3** |  |  |  |  |
| Hematoma | 1 (5.6) | 1 (5.6) | 0 (0.0) | 0 (0.0) |
| Hypertension | 1 (5.6) | 0 (0.0) | 1 (5.6) | 0 (0.0) |
| Hypotension | 3 (16.7) | 3 (16.7) | 0 (0.0) | 0 (0.0) |

**Supplementary Table 2.** Summary of serious adverse events in study population

| **All Serious AEs, n (%)** | **Dose Escalation (n=12)** | | **Dose Expansion Seli 80 mg/wk (n=6)** | **All Patients**  **(n=18)** |
| --- | --- | --- | --- | --- |
|  | **Seli 60 mg/wk (n=7)** | **Seli 80 mg/wk (n=5)** |  |  |
| Febrile neutropenia | 3 (43%) | 0 | 1 (16%) | 4 (22%) |
| Fever | 0 | 0 | 1 (16%) | 1 (5%) |
| Sepsis | 1 (14%) | 1 (20%) | 0 | 2 (10%) |
| Epistaxis | 0 | 0 | 1 (16%) | 1 (5%) |
| ICH | 1 (14%) | 0 | 0 | 1 (5%) |
| Bone pain | 1 (14%) | 0 | 0 | 1 (5%) |
| Fatigue | 0 | 0 | 1 (16%) | 1 (5%) |
| Rash | 1 (14%) | 0 | 0 | 1 (5%) |
| Hyponatremia | 0 | 0 | 1 (16%) | 1 (5%) |
| Vertigo | 0 | 1 (20%) | 0 | 1 (5%) |
| Lung infection | 0 | 1 (20%) | 0 | 1 (5%) |
| Upper respiratory tract infection | 0 | 0 | 1 (16%) | 1 (5%) |
| Hypoxia | 0 | 0 | 1 (16%) | 1 (5%) |
| Hip fracture | 0 | 0 | 1 (16%) | 1 (5%) |
| COVID 19 | 0 | 1 (20%) | 0 | 1 (5%) |
| Disease progression | 1 (14%) | 0 | 1 (16%) | 2 (10%) |
